# Cardiorespiratory Fitness, Physical Activity, Sedentary Time and Its Association with the Atherogenic Index of Plasma in Chilean Adults: Influence of the Waist Circumference to Height Ratio

**DOI:** 10.3390/nu12051250

**Published:** 2020-04-28

**Authors:** Waleska Reyes-Ferrada, Patricio Solis-Urra, Julio Plaza-Díaz, Kabir P. Sadarangani, Gerson Luis de Moraes Ferrari, Fernando Rodríguez-Rodríguez, Carlos Cristi-Montero

**Affiliations:** 1 Escuela de Kinesiología, Facultad de ciencias de la rehabilitación, Universidad Andres Bello, Viña del Mar 2531015, Chile; 2 IRyS Research Group, School of Physical Education, Pontificia Universidad Católica de Valparaíso, Valparaiso 2374631, Chile; 3 PROFITH “PROmoting FITness and Health through Physical Activity” Research Group, Sport and Health University Research Institute (iMUDS), Department of Physical Education and Sports, Faculty of Sport Sciences, University of Granada, 18071 Granada, Spain; 4 Institute of Nutrition and Food Technology “José Mataix”, Center of Biomedical Research, University of Granada, Avda. del Conocimiento s/n. Armilla, 18016 Granada, Spain; 5 Department of Biochemistry and Molecular Biology II, School of Pharmacy, University of Granada, 18071 Granada, Spain; 6 Instituto de Investigación Biosanitaria IBS.GRANADA, Complejo Hospitalario Universitario de Granada, 18014 Granada, Spain; 7 School of Physiotherapy, Faculty of Health Sciences, Universidad San Sebastian, Santiago 7510157, Chile; 8 Escuela de Kinesiología, Facultad de Salud y Odontología, Universidad Diego Portales, Santiago 8370057, Chile; 9 Laboratorio de Ciencias de la Actividad Física, el Deporte y la Salud, Facultad de Ciencias Médicas, Universidad de Santiago de Chile, USACH, Santiago 7500618, Chile

**Keywords:** adiposity, cardiovascular risk, epidemiology, healthy lifestyles, lipid profile

## Abstract

Atherogenic index of plasma (AIP) is a novel biomarker related to cardiovascular disease (CVD). Cardiorespiratory fitness (CRF) and physical activity (PA) have an inverse relationship with the AIP, while sedentary time (ST) and fatness present a positive association. This study aimed to determine the combined and independent association of CRF, PA, and ST with the AIP, and additionally to establish the waist-to-height ratio (WHtR) mediation role. Data from the Chilean national health survey were used (4671 adults). A PACS (Physical Activity Cardiorespiratory Sedentary) score was created ranging from 0 to 3, indicating the number of positive recommendations met (PA, ST, and CRF). AIP was calculated (Log10 triglycerides/high-density lipoprotein-cholesterol). The combined analysis showed that compared to those with a PACS score of 0, those with a score of 1 or 2 did not present significantly reduced AIP values (adjusted by the WHtR); however, those with a score of 3 did (OR (odds ratio) = 0.50; 95% CI, 0.32 to 0.77; *p* < 0.001). Independent analysis showed that CRF seems to be the only variable that supports the combined result (β = −0.212; *p* < 0.001). Finally, the mediation analysis indicated that the WHtR mediated the association between CRF and the AIP in 34.2% of cases. Overall, only CRF had a significant and inverse association with the AIP. Nonetheless, around one-third of this beneficial relationship is affected by an elevated WHtR.

## 1. Introduction

Cardiovascular diseases (CVD) correspond to the first cause of death in the world. It is estimated that 17.6 million people died of CVD in 2016 globally [1]. For this reason, understanding the interaction between predisposing and protective factors in CVD in addition to having simple methods that identify people at risk is fundamental to reverse this problematic situation.

CVD have both health and social impacts, hence, detecting risk factors at an early stage is vital. In this context, a novel and robust biomarker to detect CVD risk is the atherogenic index of plasma (AIP) [2,3], which correlates positively with the fractional esterification rate of high-density lipoprotein circulating (HDL-C) and inversely with the size of low-density lipoprotein circulating (LDL-C) particles [4]. This index is easily calculated from the lipid profile [5] as the logarithm of the plasma triglycerides to HDL-C ratio, which reflects the relationship between protective and atherogenic lipoproteins [3]. The AIP has been strongly associated with an elevated CVD risk than the lipid profile alone, but it has also been highly correlated with CVD risk when compared to other risk parameters (e.g., Castelli risk index or atherogenic coefficient) [6]. Thus, it can be used as an alternative and simple marker of atherogenicity.

Evidence supports that an AIP increase is associated with an augment in gamma gap (total protein (g/dL)–albumin (g/dL)) [7], risk of type 2 diabetes [8,9], high blood pressure and vascular events [8], arterial stiffness [10], non-alcoholic fatty liver disease [11], abdominal obesity [12], and the waist-to-height ratio (WHtR) [13]. Regarding the last factor, WHtR is a simple and effective anthropometric index to identify cardiometabolic risk associated with fatness. It has shown superiority over waist circumference and the body mass index (BMI) [14].

In another context, unhealthy habits, such as physical inactivity and the increase of sedentary time (ST) with the consequent low cardiorespiratory fitness (CRF), are among the major public health concerns and are considered crucial modifiable risk factors for CVD [15]. Regular physical activity (PA) and high levels of CRF have shown a strong and inverse association with the risk of CVD [16]. Collectively, Edwards et al. [17] recently reported that having favorable levels of sedentary behavior, PA, and CRF was associated with lower AIP values.

Although the AIP is considered a strong CVD predictor, there is significant variability in its values among different populations, and this discrepancy can be partly interpreted by the difference in age, region, and ethnicity [18]. In fact, scarce studies using the AIP have been conducted in Latin American (LA) population. According to data from the Pan American Health Organization from 1970 to 2000, CVD mortality decreased in the United States and Canada in a substantial and sustained manner, while in the LA, this decrease was less pronounced. An explanation for this phenomenon is the high prevalence of CVD risk factors (obesity, physical inactivity, smoking, and dyslipidemias) among low- and middle-income countries [19,20]. Therefore, the main objective of this study was to determine the combined and independent association of CRF, ST, and PA with AIP in a representative Chilean sample, and additionally to observe the influence of the WHtR as an essential variable linked to fatness.

## 2. Materials and Methods

### 2.1. Design and Participants

The database used in this study was extracted from Chilean national health survey 2016–2017 (CNHS 2016–2017) conducted between August 2016–March 2017 [21], and it was provided by the Department of Epidemiology of the Ministry of Health of Chile. CNHS 2016–2017 is a cross-sectional prevalence study conducted in homes, probabilistic, stratified by conglomerates, and multistage that considered 6233 subjects older than 15 years, with national, regional, and urban/rural representation [21]. The sample size was calculated with a relative sampling error of less than 30% and an absolute sampling error of 2.6% to the national level. The participation rate was 90.2%. The participants signed informed consent, and the study protocol was approved the Ethics Research Committee of the Faculty of Medicine at the Pontifica Universidad Católica de Chile.

### 2.2. Participants’ Evaluation

Standardized protocols were used, and all investigators (nurses and research technicians) underwent training sessions before the implementation of the survey.

### 2.3. Anthropometric Measurements

Body weight, height, and waist circumference were measured using standardized protocols described extensively in CNHS 2016–2017 [21]. Briefly, waist circumference was measured in the middle axillary line, at the midpoint between the costal margin and the iliac crest, with a metric tape [21].

### 2.4. Classification of Physical Activity and Sedentary Time

Self-reported PA levels and ST were determined using the Global Physical Activity Questionnaire (GPAQ v.2), an instrument that had been previously validated in the Latin American population [22]. The PA categories were defined according to the standard criteria of the questionnaire. Those who had <600 metabolic equivalent (MET) minutes per week were considered “physically inactive,” and those who had ≥600 MET minutes per week were considered “physically active” [23].

Sedentary time was used as a proxy of sedentary behavior [24]. ST was determined as time allocated to activities that involve sitting or reclining during leisure time or work. The question was, “How much time do you usually spend sitting or reclining on a typical day (hours and minutes per day)?” Based on previous studies [25], the high (≥240 min/day) or low levels of ST (<240 min/day) were identified.

### 2.5. Classification of the Cardiorespiratory Fitness

CRF was estimated using the Myers’ equation considering age, sex, and body weight [26]. Each participant was classified into groups of high or low CRF according to the Fitness Registry and the Importance of Exercise National Database (FRIEND registry) reference values adjusted for sex and age in subgroups for each decade between 20 and 70 years [27].

### 2.6. Calculation of Physical Activity, Cardiorespiratory Fitness, and Sedentary Time Index Score (PACS)

A PACS score was created ranging from 0 to 3, indicating the number of criteria met [17]. Thus, zero points were given for not meeting any of the three criteria and one point for each criterion: (a) physically active (≥600 MET minutes per week), (b) presently, low levels of ST (<240 min/day), and (c) a CRF level greater than or equal to their reference value.

### 2.7. Atherogenic Index of Plasma (AIP)

The AIP was assessed using a blood sample and calculated by using the following equation:

Log_10_ (triglycerides (TG)/HDL-C) (1)

with TG and HDL-C, where each concentration is expressed in mmol/L. The AIP was expressed as a continuous variable and then dichotomized into elevated/non-elevated (>0.24) [7,17,28].

### 2.8. Covariates

Two questions were extracted from CNHS 2016–2017 and used as covariates: tobacco: “Do you currently smoke cigarettes?” (4 categories: (a) ≥1/day, (b) Occasionally, <1/day, (c) No, I quitted, and (d) I have never smoked); and alcohol: “How often do you drink an alcoholic beverage?” (5 categories: (a) Never, (b) 1/month, (c) 2–4/month, (d) 2–3/week, and (e) >3/week). Furthermore, the educational level was categorized as follows: (a) <8 years, (b) 8–12 years, and (c) >12 years of education.

### 2.9. Statistical Analysis

Participant characteristics are presented as the means and standard deviations (SD) and frequencies with percentages for continuous and categorical variables, respectively (Table 1). A parametric test for data with normal and non-normal distributions according to the central limit theorem was used; it indicated that this kind of test is safe with skewed data when the sample size is over 500 [29]. First, an analysis of variance (ANOVA) with the Bonferroni post-hoc test was performed to test differences of demographic characteristics between PACS groups. Second, to examine the combined association among the key variables, three models of binary logistic regressions were performed. The AIP was established as the outcome variable (elevated/non-elevated), and the PACS score as a factor (the PACS score of 0 was considered the reference group) adjusted for age and sex (Model 1). Model 2 was adjusted additionally for tobacco, alcohol consumption, and educational level, and model 3 was adjusted additionally for the WHtR. Third, to rationalize the mediation analysis, a simple linear regression analysis was performed using the AIP score as the main outcome (as a continuous variable) and each lifestyle variable as predictors (CRF, PA, and ST). Only CRF was significantly associated with the AIP score, therefore, to test the role of the WHtR (as a variable linked to fatness), a mediation analysis was performed using the AIP as the dependent variable and CRF as the independent variable. Partial correlation and multicollinearity were checked before carrying out the mediation analysis. The mediation effect of the WHtR on the association between CRF and the AIP was analyzed through bootstrapped (10,000 samples) linear regression analyses [30] using the PROCESS SPSS script [31] adjusted for age, sex, tobacco, alcohol consumption, and educational level.

The model included three equations; equation 1 regressed the mediator (WHtR) on the independent variable (CRF); equation 2 regressed the outcome variable (AIP) on the independent variable (CRF); equation 3 regressed the outcome variable (AIP) on both the independent (CRF) and the mediator variables (WHtR). The percentage of the total effect that is accounted for by mediation was calculated using the standardized coefficients as follows: equation (1) × (2) / (3). A significant “indirect effect” (mediation) was established when (a) the independent variable was significantly related to the mediator, (b) the independent variable was significantly related to the dependent variable, (c) the mediator was significantly related to the dependent variable, and (d) the association between the independent and the dependent variable (“direct effect”) was attenuated when the mediator was included in the regression model [32]. The Sobel test was carried out to assess the strength of the “indirect effect” of the independent variable on the dependent variable to the point null hypothesis that it equals zero [33]. Point estimates and confidence intervals (CI) 95% were estimated for the indirect effect. Complete mediation was established when the independent variable (CRF) was not associated with the AIP after the mediator (WHtR) had been controlled, making path 3’ zero. While the partial mediation was established when the path from CRF to the AIP is reduced, but it is still different from zero when the mediator is introduced. All analyses were completed using IBM SPSS Statistics v 21.0 (IBM Corp, Armonk, NY, USA). The level of significance was set at *p* < 0.05.

## 3. Results

Of the total sample included in CNHS 2016–2017 (*n* = 6233), 74.93% (*n* = 4671) had information regarding ST, PA, CRF, and the AIP, with ages between 20 and 70 years. Table 1 shows characteristics of the sample as divided into groups by the PACS score. Women amount to 64.2% of the sample, and it can be observed that most health variables are better if participants fulfill more recommendations (PACS) in a dose–response manner.

### 3.1. Models for Combined Analysis

**Figure 1:**
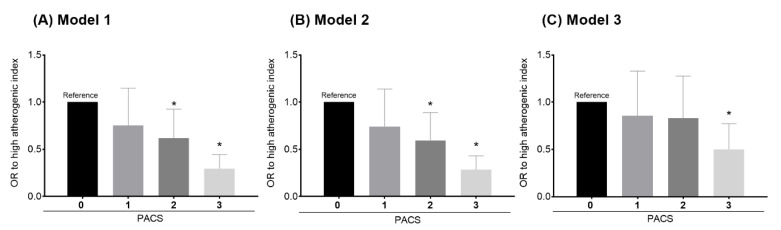
(**A**) Model 1 shows the binary logistic regression PACS score (number of met recommendations) for the Aherogenic Index AIP (elevated/non-elevated) adjusted for age and sex. (**B**) Model 2 is adjusted for model 1 plus tobacco, alcohol consumption, and educational level. (**C**) Model 3 is adjusted model 2 plus the Waist Circumference to Height Ratio WHtR. OR: odds ratio. Error bars represent the confidence interval (95% CI). * *p*-value < 0.05.

Figure 1 shows that the odds of presenting an elevated AIP depends on the number of recommendations fulfilled according to the 3 models analyzed.

Model 1 showed that compared to those with a PACS score of 0, those with a score of 1, 2, or 3 present a reduction of odds of an elevated AIP (OR (odds ratio) = 0.75; 95% CI, 0.49 to 1.14; *p* = 0.18, OR = 0.61; 95% CI, 0.41 to 0.92; *p* = 0.020, and OR = 0.29; 95% CI, 0,19 to 0.44; *p* < 0.001, respectively). Model 2 showed that compared to those with a PACS score of 0, those with a score of 1, 2, and 3 present a reduction of odds of an elevated AIP (OR = 0.74; 95% CI, 0.48 to 1.13; *p* = 0.172, OR = 0.59; 95% CI, 0.39 to 0.88; *p* = 0.012, and OR = 0.28; 95% CI, 0,18 to 0.43; *p* < 0.001, respectively). And finally, model 3 showed that compared to those with a PACS score of 0, those with a score of 1, 2, and 3 present a reduction of odds of an elevated AIP (OR = 0.85; 95% CI, 0.55 to 1.32; *p* = 0.48, OR = 0.83; 95% CI, 0.54 to 1.27; *p* = 0.39, and OR = 0.50; 95% CI, 0.32 to 0.77; *p* < 0.002, respectively).

### 3.2. Independent Analysis

Simple linear regression analyses showed that while ST was not associated with the AIP (β = −0.011; *p* = 0.544), both PA and CRF showed a borderline and significant negative association with the AIP, respectively (β = −0.033; *p* = 0.062; and β = −0.212; *p* < 0.001). Then, as only CRF presented a significant statistical association with AIP values, ST and PA were excluded from the mediation analysis.

Partial correlation coefficients between the WHtR, CRF, and the AIP are presented in Table 2. No sex-related impact was observed (*p* = 0.160). CRF was negatively associated with the AIP as well as with the WHtR (*p* < 0.001), while the WHtR was positively associated with the AIP (*p* < 0.001).

**Figure 2:**
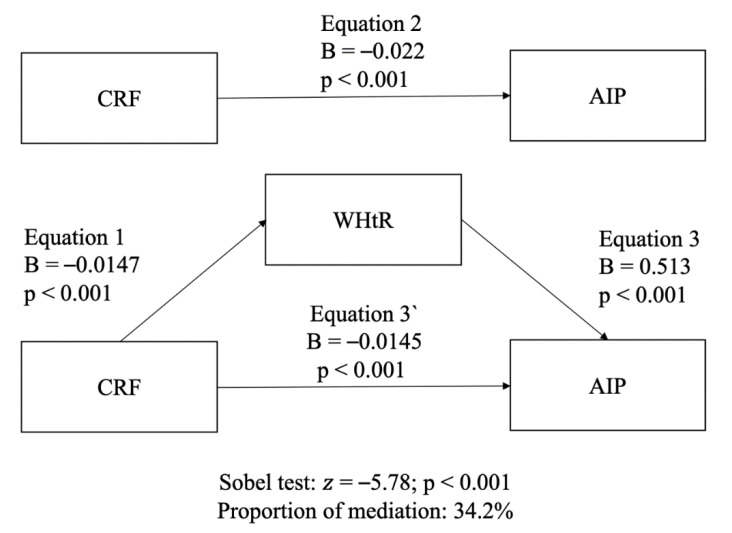
The mediation effect of the WHtR on the association between CRF and the AIP adjusted for potential confounders (age, sex, tobacco, alcohol consumption, and educational level). CRF: cardiorespiratory fitness; WHtR: waist-to-height ratio; AIP: atherogenic index of plasma. B represents unstandardized values.

Figure 2 shows results of the mediation analysis. Overall, CRF and the AIP were significantly and inversely associated. Mediation analysis revealed that the association between CRF and the AIP was mediated by the WHtR. The role of this mediation adjusted for potential confounders accounted for around 34.2%.

## 4. Discussion

The purpose of this study was to determine the combined and independent association of CRF, PA, and ST with the AIP in a Latin American country to determine whether the sum of these health indicators reduces the odds of a high AIP level. Also, the WHtR mediation role on the association between CRF and the AIP was tested to determine how the fatness indicator affects this relationship.

On the one hand, both the independent and combined analyses showed a dose–response relationship regarding meeting recommendations (low ST, high CRF, and meeting PA guidelines) and a lower AIP. Fulfilling one, two, or three recommendations was associated with reduced odds of presenting an elevated AIP of 26%, 41%, and 71.5%, respectively. However, only significant differences were achieved when two or three recommendations were met. These results are consistent with the findings of Edwards et al., who reported a 68% decrease in the odds of having an elevated AIP when all three described parameters were met [17].

On the other hand, another interesting finding is that when the WHtR was included in model 3, the odds of presenting a lower AIP decreased by 21.5% (from 71.5% to 50%). This outcome sheds light on the importance of abdominal obesity, which reduces the beneficial relationship between PA and the AIP [11,34]. In addition to this, previous studies reported a positive correlation between the WHtR and the AIP and a negative association between the WHtR and HDL-C [12,13]. Thus, it seems to be fully justified to have investigated in this study the mediating role of the WHtR on the association between CRF and the AIP. Indeed, our findings show that the WHtR partially affected this relationship (34.2%), which gives great relevance to the protective role of CRF in the CVD risk. The aforementioned provides additional evidence concerning the “fat-but-fit” hypothesis in overweight individuals, in whom high CRF levels result in a lower all-cause mortality risk compared to normal-weight individuals with low CRF [35,36].

Besides, while there is compelling evidence about the benefits of having a high CRF level in terms of the CVD and mortality risk, even in overweight individuals, the role of fatness on the global health outcome is undeniable [12,36]. Recently, an interesting discussion has emerged to discuss whether obesity health risks have been exaggerated or not. Some researchers state that CRF is capable of attenuating or eliminating the mortality risk associated with high BMI. Moreover, the removal of body fat does not improve cardiometabolic health [37]. However, a counter-response note indicates that the health risks of obesity have not been exaggerated. These researchers suggest that for optimizing the population health, the efforts should be focused on modifying lifestyle behaviors, which influence both weight status and CRF positively [38]. Based on our findings, we agree regarding the favorable association of CRF with amelioration of the risk of elevated AIP. Nonetheless, it is also pertinent to consider that the WHtR interferes with more than a third of the relation between the WHtR and the AIP. Hence, we support the recommendations raised by Jakicic et al., which involve a more comprehensive strategy to improve the lipid profile and CV health [38].

From the public health point of view, in some measure, the present findings suggest that the more recommendations are met, the better the results. Nevertheless, among the three recommendations studied, only a higher CRF level had the strongest association with a significant reduction in the odds of having a higher AIP, even independently of the WHtR. For this reason, it has been proposed to incorporate this health indicator (CRF) as a vital sign in the CVD risk assessment in the clinical anamnesis [39].

In accordance with our results, some studies have pointed out the beneficial role of CRF in the plasma lipid levels and the risk of developing atherogenic dyslipidemia [40,41]. These valuable findings could be partially explained by the fact that higher CRF improves the oxidative metabolism in muscles, enhancing the clearance of TG, increasing HDL levels and the activity of the lipoprotein lipase. This last enzyme is responsible for hydrolyzing TG in chylomicrons and very low-density lipoproteins [40,42].

But it is important to note that to get an improvement of the CRF level, it is fundamental to reduce the time spent sitting and increase the duration and intensity of exercise [43]. On the one hand, sedentary time generates a harmful impact on CRF, even in the physically active population [44]; and on the other hand, high amounts of moderate to vigorous PA (MVPA) are necessary to eliminate the association between sedentary time and the CVD mortality risk [45]. In short, only an adequate balance between sedentary time and MVPA could trigger an improvement in CRF.

### Strengths and Limitations

Within the limitations of this study, the cross-sectional design should be considered, as it prevents determination of causality. The measurements of PA and ST were performed through a self-report questionnaire, of CRF—by an indirect equation. Besides, the reference CRF values are based on the United States population, since there are no reference tables for the Chilean population yet. However, a critical study limitation is the Myers’ equation, which estimates CRF on the basis of sex, age, and body weight. Therefore, CRF findings could be highly dependent on body weight. Regarding that, adjusting the models to body weight would result in duplication of the variable within the analysis and, therefore, in a methodological bias. Thus, we tested the association between CRF and body weight, and we found a significant relationship between them (r = 0.20; < 0.001; however, it explains only 4% of the total variance. The aforementioned indicates that, to some extent, our outcomes do not depend on body weight significantly. Furthermore, evidence showed that BMI or waist circumference can improve the CRF estimation [46]; however, our objective was to test the WHtR as a mediator, making it impossible to use an equation different from the one used in this study. Finally, we consider having used a national health survey with a representative sample the strength of this study. This feature allows us to extrapolate these results to the Chilean adult population generating non-existent information in the Latin American region.

## 5. Conclusions

In conclusion, the more recommendations a person complies with (CRF, PA, or ST), the less likely they are to have a high AIP. Among CRF, PA, and CRF, the last can be considered the most important indicator associated with optimal levels of the AIP. Still, it is also fundamental to recognize that the WHtR considerably mediates this relationship. Encouraging the population to increase their CRF, along with reducing their WHtR, could be a synergistic and comprehensive recommendation to decrease the AIP.

## Figures and Tables

**Table 1:** Characteristics of the participants as divided by the PACS score, Chilean national health survey 2016–2017 (*n* = 4671).

Variables	Overall (*n* = 4671)	PACS Score				*p*-Value
		0 (*n* = 291)	1 (*n* = 1140)	2 (*n* = 1854)	3 (*n* = 1386)	
Age (years)	49.40 ± 16.40	48.48 ± 17.36	49.14 ± 16.96	48.95 ± 16.18	50.42 ± 15.97	0.156
Sex (*n*/%)	M 1670/35.7 W 3001/64.2	M 95/32.6 W 196/67.4	M 339/29.7 W 801/70.3	M 636/34.3 W 1218/65.7	M 600/43.3 W 786/56.7	0.000
Body mass (kg)	74.90 ± 15.35	89.61 ± 14.30	80.25 ± 16.03	75.04 ± 15.26	67.24 ± 10.05	0.000
Height (cm)	159.93 ± 9.30	161.76 ± 10.17	159.96 ± 9.37	159.98 ± 9.30	159.46 ± 9.00	0.076
Body mass index (kg/m^2^)	29.28 ± 5.49	34.33 ± 5.20	31.39 ± 5.98	29.29 ± 5.31	26.44 ± 3.39	0.000
Atherogenic index of plasma (mmol/L)	0.44 ± 0.30	0.52 ± 0.30	0.48 ± 0.29	0.45 ± 0.29	0.38 ± 0.30	0.000
% (*n*) meeting PA guidelines	30.4% (1,424)	0% (0)	5.7% (266)	24.7% (1,158)	100% (1,386)	0.000
Cardiorespiratory fitness (mL/kg/min)	30.86 ± 9.08	26.67 ± 8.96	28.63 ± 9.29	30.79 ± 8.83	33.63 ± 8.39	0.000
Sedentary time (min/day)	178.20 ± 176.10	404.32 ± 173.01	271.77 ± 212.07	153.74 ± 151.86	86.48 ± 57.39	0.000
Waist circumference (cm)	94.80 ± 13.63	105.88 ± 12.84	99.35 ± 13.88	94.76 ± 13.35	88.76 ± 10.73	0.000
Waist-to-height ratio	0.60 ± 0.09	0.65 ± 0.09	0.62 ± 0.09	0.59 ± 0.08	0.55 ± 0.07	0.000
Tobacco (*n*/%)						0.387
≥1/day	1085/23.2	70/24.1	258/22.6	417/22.5	340/24.5	
Occasionally, < 1/day	326/7.0	20/6.9	72/6.3	119/6.4	115/8.3	
No, I quitted	1148/24.6	73/25.1	285/25	454/24.5	336/24.2	
I have never smoked	2112/45.2	128/44	525/46.1	864/46.6	595/42.9	
Alcohol (*n*/%)						0.001
Never	1593/34.1	95/32.6	414/36.3	649/35	435/31.4	
1/month	1738/37.2	121/41.6	419/36.8	700/37.8	498/35.9	
2–4/month	984/21.0	55/18.9	233/20.4	380/20.5	316/22.8	
2–3/week	241/5.2	12/4.1	42/3.7	96/5.2	91/6.6	
>3/week	114/2.4	8/2.7	32/2.8	28/1.5	46/3.3	
Educational level (*n*/%)						0.000
<8 years	1064/22.8	66/22.7	271/23.8	432/23.3	295/21.3	
8–12 years	2465/52.8	134/46	566/49.6	965/52	800/57.7	
>12 years	1104/23.7	90/30.9	300/26.3	434/23.4	280/20.2	

Values are mean ± standard deviation or frequency (% [*n*]); M: men; W: women; PA: physical activity; PACS: physical activity cardiorespiratory, sedentary. *p*-value < 0.05 represents a significant main difference between groups.

**Table 2:** Partial correlation coefficients between the AIP, CRF, and the WHtR, Chilean national health survey 2016-2017 (*n* = 4671).

	CRF	WHtR	AIP
CRF	-	-	-
WHtR	−0.722 *	-	-
AIP	−0.306 *	0.297 *	-

CRF: cardiorespiratory fitness; WHtR: waist-to-height ratio: AIP: atherogenic index of plasma. Model adjusted for age, sex, tobacco, alcohol consumption, and educational level. * Significant association (*p* < 0.001).
